# A Radish Basic Helix-Loop-Helix Transcription Factor, RsTT8 Acts a Positive Regulator for Anthocyanin Biosynthesis

**DOI:** 10.3389/fpls.2017.01917

**Published:** 2017-11-08

**Authors:** Sun-Hyung Lim, Da-Hye Kim, Jae K. Kim, Jong-Yeol Lee, Sun-Hwa Ha

**Affiliations:** ^1^Department of Agricultural Biotechnology, National Institute of Agricultural Science, Jeonju, South Korea; ^2^Division of Life Sciences and Bio-Resource and Environmental Center, Incheon National University, Incheon, South Korea; ^3^Department of Genetic Engineering and Graduate School of Biotechnology, Kyung Hee University, Yongin, South Korea

**Keywords:** anthocyanin, bHLH, MYB, radish, transcription factor

## Abstract

The MYB-bHLH-WDR (MBW) complex activates anthocyanin biosynthesis through the transcriptional regulation. RsMYB1 has been identified as a key player in anthocyanin biosynthesis in red radish (*Raphanus sativus* L.), but its partner bHLH transcription factor (TF) remains to be determined. In this study, we isolated a bHLH TF gene from red radish. Phylogenetic analysis indicated that this gene belongs to the TT8 clade of the IIIF subgroup of bHLH TFs, and we thus designated this gene *RsTT8*. Subcellular localization analysis showed that RsTT8-sGFP was localized to the nuclei of *Arabidopsis thaliana* protoplasts harboring the *RsTT8-sGFP* construct. We evaluated anthocyanin biosynthesis and *RsTT8* expression levels in three radish varieties (N, C, and D) that display different red phenotypes in the leaves, root flesh, and root skins. The root flesh of the C variety and the leaves and skins of the D variety exhibit intense red pigmentation; in these tissues, RsTT8 expression showed totally positive association with the expression of RsMYB1 TF and of five of eight tested anthocyanin biosynthesis genes (i.e., *RsCHS, RsCHI, RsF3H, RsDFR*, and *RsANS*). Heterologous co-expression of both *RsTT8* and *RsMYB1* in tobacco leaves dramatically increased the expression of endogenous anthocyanin biosynthesis genes and anthocyanin accumulation. Furthermore, a yeast two-hybrid assay showed that RsTT8 interacts with RsMYB1 at the MYB-interacting region (MIR), and a transient transactivation assay indicated that RsTT8 activates the *RsCHS* and *RsDFR* promoters when co-expressed with RsMYB1. Complementation of the Arabidopsis *tt8-1* mutant, which lacks red pigmentation in the leaves and seeds, with *RsTT8* restored red pigmentation, and resulted in high anthocyanin and proanthocyanidin contents in the leaves and seeds, respectively. Together, these results show that RsTT8 functions as a regulatory partner with RsMYB1 during anthocyanin biosynthesis.

## Introduction

Radishes (*Raphanus sativus* L.) belong to Brassicaceae family and are economically important vegetable crops cultivated for producing seed oil and sprouts, as well as edible taproots. Moreover, they are good model crops for deciphering the anthocyanin biosynthesis mechanisms because they have variety of pigmentation pattern and intensity depending on the anthocyanin accumulation in leaves, stems and roots. Additionally, anthocyanin accumulated red radishes have attracted attention as potential economic sources for the natural food colorant (Jing et al., 2012).

Anthocyanins and proanthocyanidins (PAs, also called condensed tannins) are the major pigment metabolites of flavonoid compounds, and are abundant in the seed coats, leaves, fruits, flowers, and bark of many plant species (Dixon et al., 2005). The anthocyanin and flavonoid biosynthetic pathways have been extensively characterized, and the corresponding structural genes involved in these pathways have been studied in several plant species including *Arabidopsis thaliana, Oryza sativa* (rice), and *Malus domestica* (apple) (Grotewold, 2006; Lin-Wang et al., 2010; Lim and Ha, 2013). The structural genes encoding enzymes in the anthocyanin biosynthetic pathway are regulated by transcription factors (TFs) and are expressed synergistically during anthocyanin accumulation (Koes et al., 2005; Grotewold, 2006).

Anthocyanin biosynthesis is activated by the R2R3 MYB, basic helix-loop-helix (bHLH), and WD40 repeat (WDR) TFs, which form the MBW complex (Hichri et al., 2011; Xu et al., 2015). R2R3 MYB proteins in the MBW complex have a crucial role in controlling the spatio-temporal expression of anthocyanin biosynthetic genes. The R3 region of the R2R3 MYB TF interacts with the N-terminal domain of bHLH. Whereas R2R3 MYB and bHLH regulate the expression of anthocyanin biosynthesis genes, WDR forms a docking platform for bHLH (Hichri et al., 2011).

In our previous studies, RsMYB1 was verified as a positive regulator to transcriptionally activate anthocyanin biosynthetic pathway in red radish (*Raphanus sativus* L.) (Lim et al., 2016a). Transient heterologous coexpression of RsMYB1 and B-Peru [a bHLH protein known to regulate anthocyanin biosynthesis in maize (*Zea mays*)] in tobacco leaves led to synergistic accumulation of anthocyanins and ectopic expression of RsMYB1 in Arabidopsis induced the higher transcript level of endogenous bHLH genes. This result suggests that RsMYB1 controls anthocyanin biosynthesis together with its binding partner bHLH. As the identity of this bHLH protein is unknown, we sought to identify the anthocyanin-related bHLH in radish in this study.

A recent study reported that RsMYB1 activates anthocyanin biosynthesis in red radish (*Raphanus sativus* L.), and the transcript patterns of *RsMYB1* matched to those of RsTT8 in the anthocyanin accumulating tissues (Lim et al., 2016a). Transient heterologous coexpression of *RsMYB1* and *B-Peru* [a bHLH protein known to regulate anthocyanin biosynthesis in maize (*Zea mays*)] in tobacco leaves led to synergistic accumulation of anthocyanins. Thus, RsMYB1 controls anthocyanin biosynthesis together with its binding partner bHLH. As the identity of this bHLH protein is unknown, we sought to identify the anthocyanin-related bHLH in radish in this study.

bHLH proteins are a large class of transcription factors that have been assigned to 26 subgroups (Pires and Dolan, 2009). Flavonoid-related bHLHs, which cluster into subgroup IIIf, contain a MYB-interaction region (MIR) at the N-terminus, followed by a WDR interacting region through the acidic domain (WD/AD), a bHLH domain and C-terminal region, which mediate the formation of homodimers or heterodimers with other bHLH proteins (Heim et al., 2003; Feller et al., 2006). The first bHLH TFs found to regulate anthocyanin biosynthesis were identified in maize and were designated as Red1 and Booster1 (Chandler et al., 1989). Subsequent work identified a number of additional bHLH TFs that regulate flavonoid biosynthesis, including AN1 and JAF13 in petunia (*Petunia hybrida*) (Quattrocchio et al., 1998; Spelt et al., 2000), TT8 and GLABRA3 (GL3) in Arabidopsis (Nesi et al., 2000; Feyissa et al., 2009), StbHLH1 and StJAF13 in potato (*Solanum tuberosum*) (Payyavula et al., 2013; D'amelia et al., 2014), CmbHLH2 in chrysanthemum (*Chrysanthemum morifolium*) (Xiang et al., 2015), MdbHLH3 and MdbHLH33 in apple (Espley et al., 2007), and FhTT8 and FhGL3 in freesia (*Freesia hybrida*) (Li et al., 2016). Many of these bHLH TFs have been shown to regulate physiological and morphological events such as flavonoid biosynthesis and the formation of root hairs and trichomes (Grotewold, 2006). These combined results suggest that bHLH TFs act together with R2R3 MYB have a crucial role in anthocyanin biosynthesis in plants.

Here, we isolated a bHLH transcription factor in red radish, which we designated as RsTT8, and investigated its function in anthocyanin biosynthesis. We found that RsTT8 is a positive regulator of anthocyanin biosynthesis. We examined its ectopic expression in tobacco and Arabidopsis via transient and stable transformation, respectively. Although heterologous expression of *RsTT8* in tobacco leaves did not result in anthocyanin accumulation, co-expression of *RsTT8* with *RsMYB1* led to higher anthocyanin levels than expression of *RsMYB1* alone. Yeast two-hybrid (Y2H) analysis confirmed that RsTT8 interacts with RsMYB1, which is known play an important role in anthocyanin biosynthesis in radish, thus supporting its role in anthocyanin biosynthesis. In addition, a transient transactivation assay indicated that RsTT8 and RsMYB1 together activate the promoter of *RsCHS* and *RsDFR*. Furthermore, RsTT8 complemented the Arabidopsis *tt8-1* mutant, restoring anthocyanin accumulation in rosette leaves and PA accumulation in seeds. Together, these results suggest that RsTT8 mediates PA biosynthesis in seeds and interacts with RsMYB1 to mediate anthocyanin biosynthesis in leaves.

## Results

### Isolation of RsTT8 cDNA and phylogenetic analysis

To investigate the mechanism regulating anthocyanin biosynthesis in red radish, we cloned a bHLH-type TF gene from radish leaves was cloned using degenerative PCR, 5′-RACE, and 3′-RACE and designated this gene as *RsTT8. RsTT8* has an ORF of 1,560 bp encoding a polypeptide of 519 amino acids (GenBank accession number KY651179). RsTT8 contains several domains that are conserved in flavonoid-related bHLH-type TFs, including an N-terminal MIR domain, a WD/AD domain, a basic helix-loop-helix (bHLH) domain, and a C-terminal aspartokinase, chorismate mutase, TyrA (ACT)-like domain. The bHLH domain contains nearly 60 amino acids involved in DNA binding; among these residues, 19 amino acid residues are conserved in anthocyanin-related bHLH TFs (Supplementary Figure 2).

**Figure 1:**
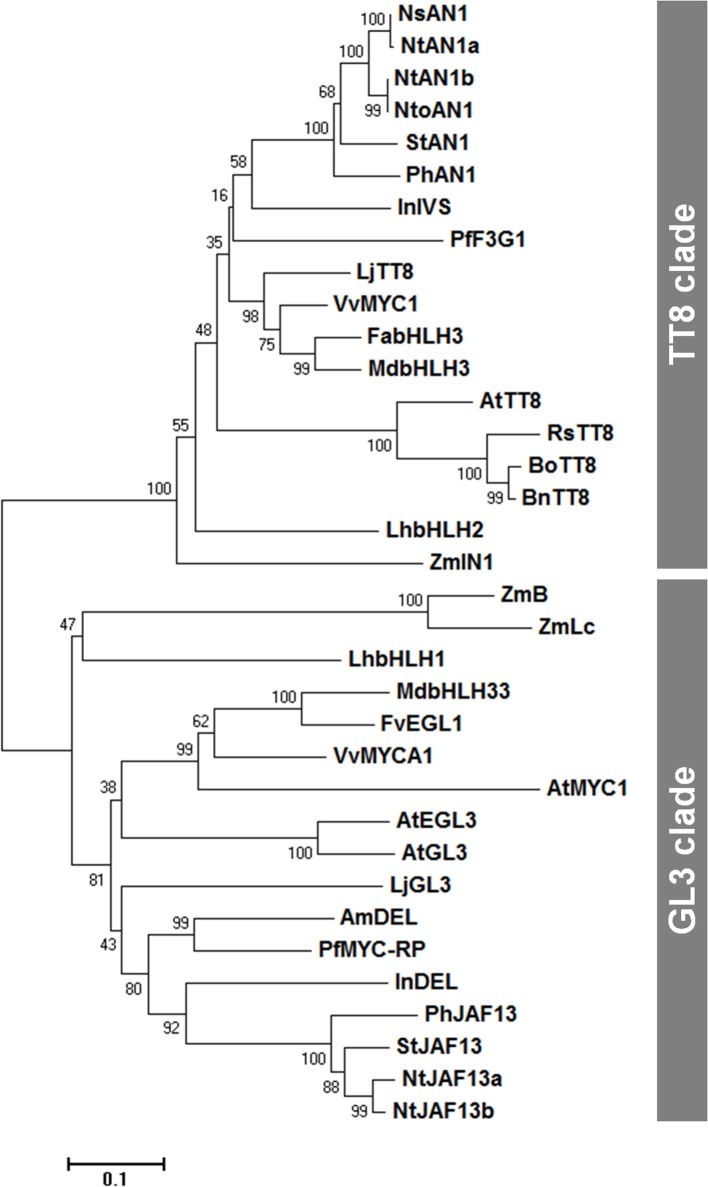
A neighbor-joining phylogenetic tree of plant bHLH IIIf TF sequences. Numbers next to the nodes are bootstrap values from 1,000 replications. The tree is drawn to scale, with branch lengths in the same units as those of the evolutionary distances that were used to infer the phylogenetic tree (scale bar, 0.1 amino acid substitutions per site). The following deduced amino acid sequences were retrieved from DDBJ/EMBL/GenBank databases: AmDEL (AAA32663) in *Antirrhinum majus*; AtEGL3 (Q9CAD0), AtGL3 (NP_680372), AtMYC1 (Q8W2F1), and AtTT8 (Q9FT81) in *Arabidopsis thaliana*; BnTT8 (NP_001302903) in *Brassica napus*; BoTT8 (ADP76654) in *Brassica oleracea*; FabHLH3 (AFL02463) in *Fragaria* × *ananassa*; FvEGL1 (XP_004308377) in *Fragaria vesca*; InDEL (BAE94393) and InIVS (BAE94394) in *Ipomoea nil*; LhbHLH1 (BAE20057) and LhbHLH2 (BAE20058) in *Lilium hybrid*; LjGL3 (AB492284) and LjTT8 (AB490778) in *Lotus japonicus*; MdbHLH3 (ADL36597) and MdbHLH33 (ABB84474) in *Malus domestica*; NsAN1 (HQ589210) in *Nicotiana sylvestris*; NtAN1a (HQ589208), NtAN1b (HQ589209), NtJAF13a (KF305768), and NtJAF13b (KF298397) in *Nicotiana tabacum*; NtoAN1 (HQ589211) in *Nicotiana tomentosiformis*; PhAN1 (AAG25928) and PhJAF13 (AAC39455) in *Petunia* × *hybrida*; PfF3G1 (AB103172) and MYC-RP (AB024050) in *Perilla frutescens*; RsTT8 (KY651179) in *Raphanus sativus*; StAN1 (JX848660) and StJAF13 (NM_001288203) in *Solanum tuberosum*; VvMYC1 (ACC68685) and VvMYCA1 (ABM92332) in *Vitis vinifera*; ZmB (CAA40544), ZmIN1 (AAB03841), and ZmLc (P13526) in *Zea mays*.

We then conducted a phylogenetic analysis of flavonoid-related bHLH group IIIf proteins, from various plant species (Figure 1), and found that these proteins cluster into two clades, the TT8 and GL3 clades, as previously described (Davies et al., 2012). RsTT8 belongs to the TT8 clade, which includes AN1 (petunia), Intensifier (maize), and TT8 (Arabidopsis). The GL3 clade contains JAF13 (petunia), R (maize), and GL3/EGL3 (Arabidopsis). Each clade of the bHLH group IIIf TFs has a unique role and/or partially redundant function (Hichri et al., 2011). For example, AtTT8 and AtGL3 have distinct functions in PA biosynthesis and trichome formation, respectively, but have partially redundant functions in anthocyanin biosynthesis.

### Subcellular localization of RsTT8

**Figure 2:**
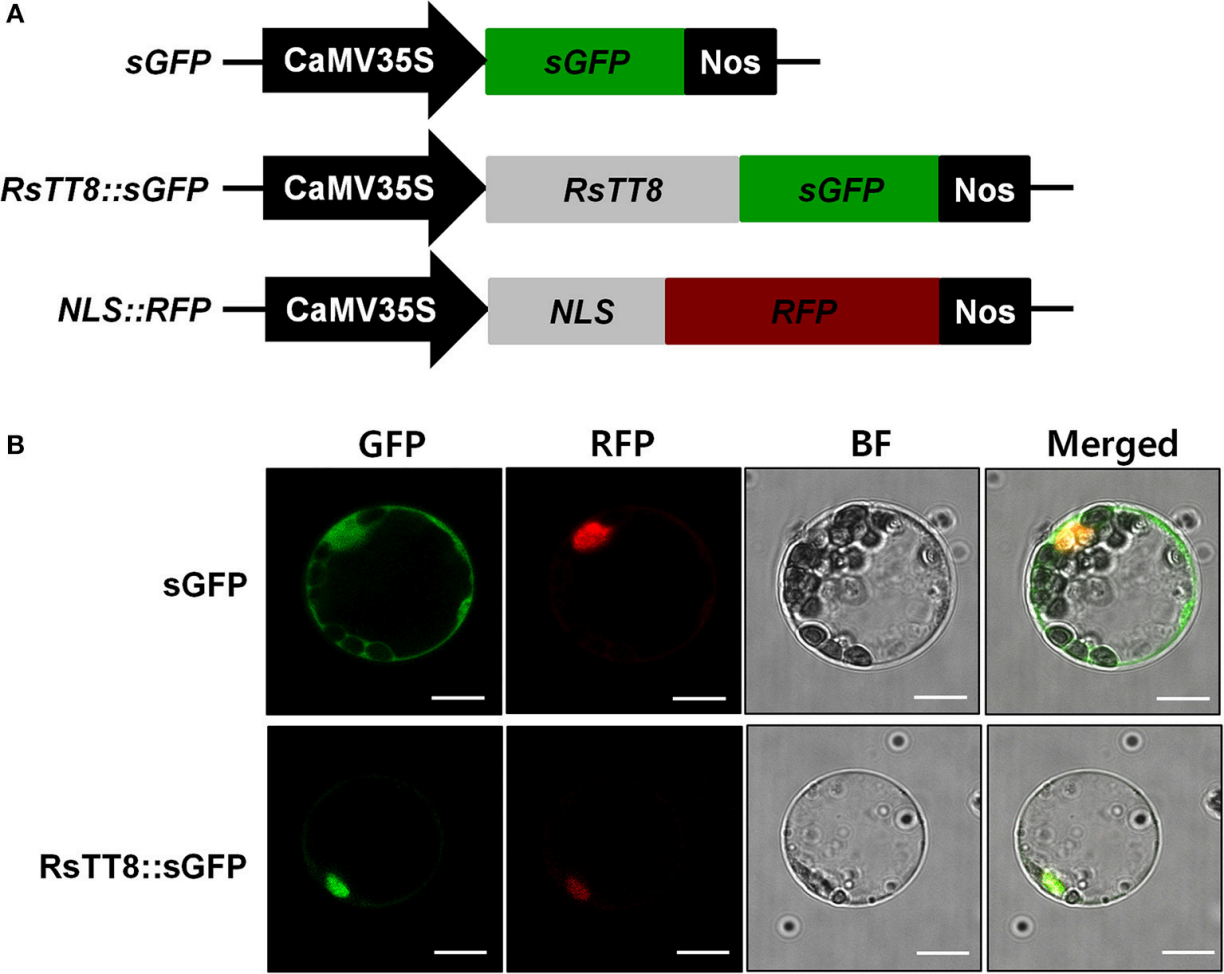
Subcellular localization of RsTT8 in Arabidopsis leaf protoplasts. **(A)** Three constructs were used in this experiment: sGFP, soluble GFP; RsTT8::GFP, RsTT8 fused to GFP; and NLS::RFP, nuclear localization signal fused with RFP. **(B)** *In vivo* targeting of RsTT8 in Arabidopsis protoplasts. Data are representative of protoplasts expressing fusion proteins at 16 h after transformation. Bar = 10 μm.

To investigate the role of RsTT8 in transcriptional regulation, we examined the subcellular localization of RsTT8 in Arabidopsis leaf protoplasts. Protoplasts were cotransformed with constructs harboring red fluorescent protein (RFP) fused to the nuclear localization signal (NLS) of the SV40 large T antigen and soluble GFP (sGFP) or a RsTT8::GFP fusion. As shown in Figure 2, GFP fluorescence was observed in the nuclei of protoplasts harboring RsTT8::GFP, but throughout the cytoplasm in those harboring the GFP control. These results indicate that RsTT8 is localized in the nucleus, which is consistent with its putative role as a transcription factor.

### Expression analysis of structural and regulatory genes in anthocyanin biosynthesis pathways of different radish cultivars

**Figure 3:**
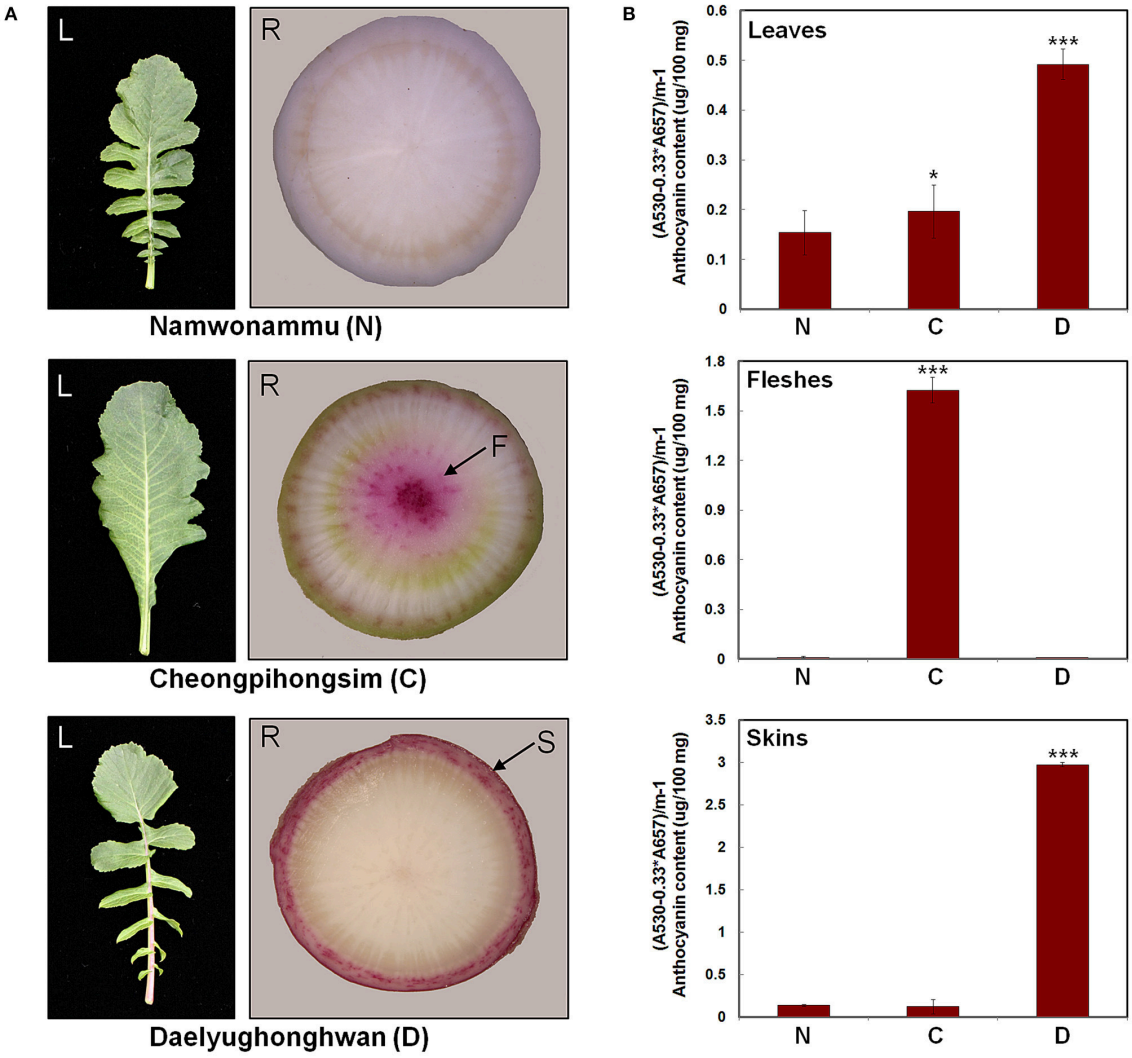
Phenotypes and anthocyanin contents in three different radish cultivars. **(A)** Leaves and roots of three radish cultivars used in this study. **(B)** Anthocyanin contents. Results represent mean values ± SD from three biological replicates. ^*^ and ^***^ indicate values that differ significantly from the N variety at *P* < 0.05 and *P* < 0.001, respectively, according to Student's paired *t*-test.

To investigate the mechanisms controlling anthocyanin biosynthesis, we analyzed three radish cultivars that exhibit differences in pigmentation. The N cultivar has green leaves and white root flesh and skin. The C cultivar has green leaves and red root flesh with white root skin. The D cultivar has red leaves and white root flesh with red root skin (Figure 3A). Anthocyanin contents were quantified in the leaves, root flesh, and root skin of these three radish cultivars (Figure 3B). Anthocyanin contents were essentially consistent with the visible red pigmentation; anthocyanin levels were high in the leaves of the D cultivar, flesh of the C cultivar, and skin of the D cultivar. These results suggest that differences in the anthocyanin contents of leaves, root flesh, and root skin are responsible for differences in the red-coloration phenotypes of the radish cultivars.

**Figure 4:**
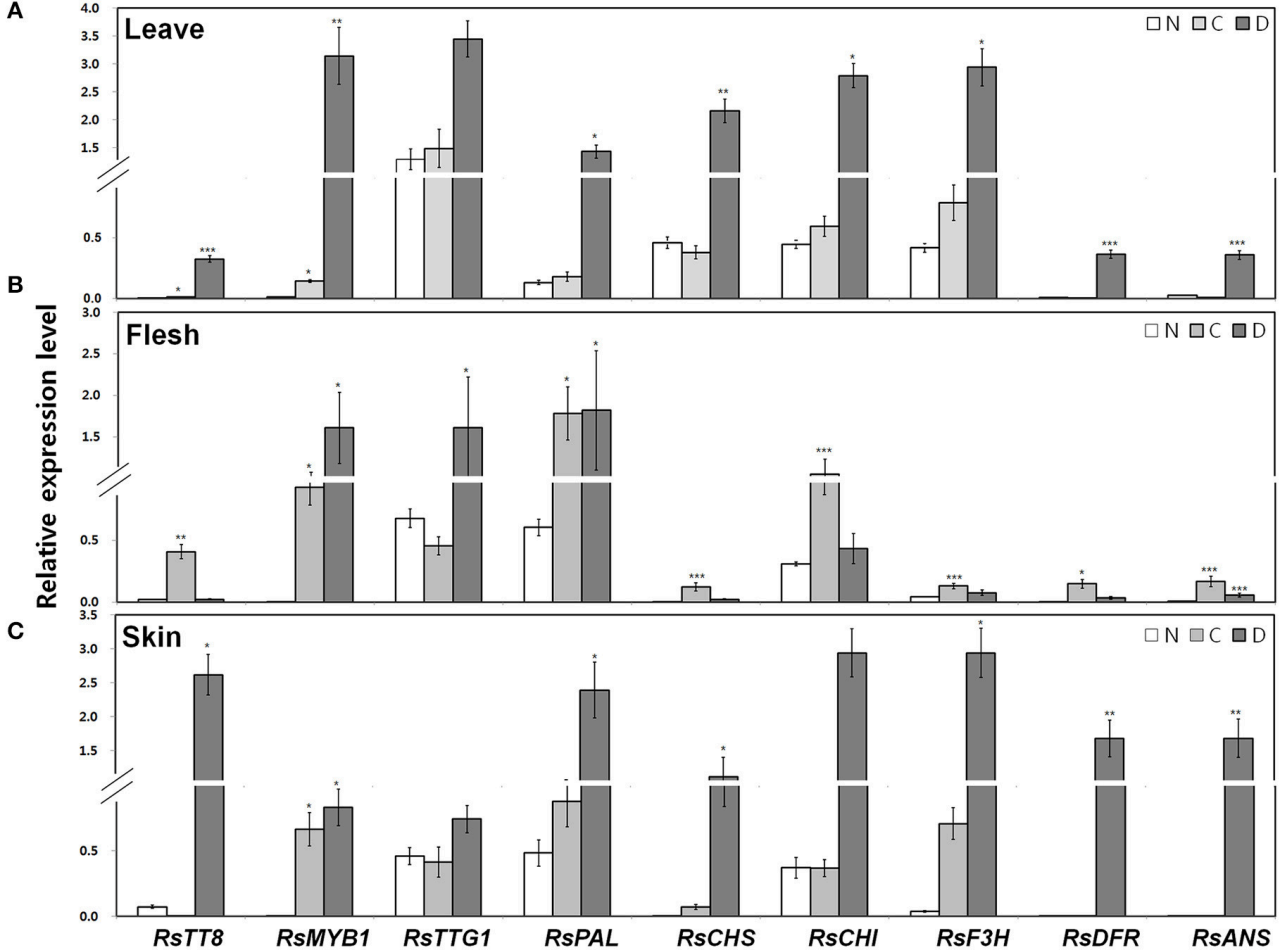
Expression of regulatory and structural genes in the anthocyanin biosynthesis pathway in three different radish cultivars. qPCR analysis of anthocyanin biosynthesis genes inleaves **(A)** root flesh **(B)**, and root skin **(C)**. Results represent mean values ± SD from three biological replicates. ^*^, ^**^, and ^***^ indicate values that differ significantly from the N variety at *P* < 0.05, *P* < 0.01, and *P* < 0.001, respectively, according to Student's paired *t*-test.

Next, we examined the transcript levels of the following genes in the leaves, root flesh, and root skin of the three radish cultivars (Figure 4): three regulatory genes, including the bHLH-type *RsTT8*, the R2R3-MYB-type *RsMYB1*, and the WDR *RsTTG1*; and six structural genes involved in anthocyanin biosynthesis, including the upstream gene *RsPAL*, the early biosynthetic genes (EBGs) *RsCHS, RsCHI*, and *RsF3H*, and the late biosynthetic genes (LBGs) *RsDFR* and *RsANS*. The transcript levels of *RsTT8* and *RsMYB1* were higher in red leaves (i.e., the D variety) than in green leaves (the C and N varieties). Similar to the expression pattern of *RsTT8* and *RsMYB1*, the transcript levels of all structural genes were higher in the D cultivar than in the C and N cultivars (Figure 4A). Particularly, the higher expression levels of *RsMYB1* and *RsTT8* in red leaves (the D variety) than in green leaves (the C and N varieties) were similar to those of the two LBGs. *RsTT8* and *RsMYB1* expression levels were higher in red root flesh (the C variety) than in white root flesh (the D and N varieties), similar to those of all structural genes (Figure 4B). Interestingly, the highest expression levels of *RsMYB1* and *RsTTG1* were detected in white root flesh (the D variety), whereas the transcript levels of five structural genes (three EBGs and two LBGs, excluding *RsPAL*) were lower in white (the D variety) than in red (the C variety) root flesh. The lowest expression levels of *RsTT8* and *RsMYB1* were detected in white root skin (the N variety), and these levels were significantly associated with the levels of *RsCHS* and the two LBGs. In the root skin also, the transcript levels of all six structural genes were significantly matched to those of *RsTT8* and *RsMYB1* in red root skin (the D variety) (Figure 4C). Interestingly, the transcript level of *RsTT8* was low in white root skin (the C variety), whereas the transcript level of *RsMYB1* was high. As for the *RsTT8* expression pattern, transcripts of two EBGs (*RsCHS* and *RsF3H*) and the two LBGs were barely detected in white root skin (the C variety). The transcript levels of two LBGs were significantly associated with those of *RsTT8* and *RsMYB1* in white root skin (the N variety). These combined results indicate that high expression levels of *RsTT8* and *RsMYB1* are associated with high levels of *RsCHS, RsDFR* and *RsANS* transcript and anthocyanin content in all tested organs of the three radish cultivars (Figures 3, 4).

### Transient coexpression of RsTT8 and RsMYB1 enhances anthocyanin accumulation in tobacco leaves

**Figure 5:**
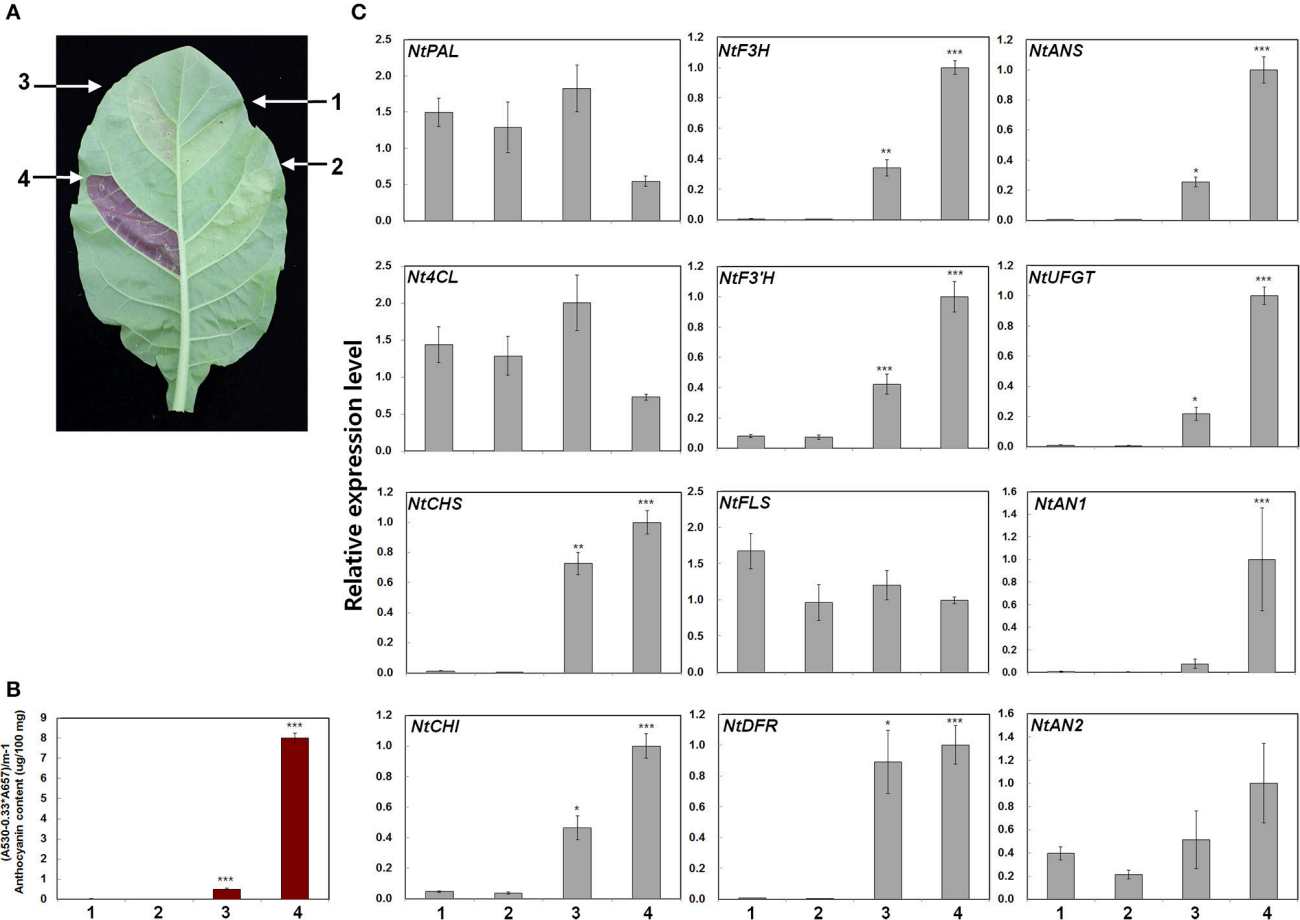
Anthocyanin contents in transiently transformed tobacco leaves infiltrated with Agrobacterium strains carrying *RsMYB1* and *RsTT8*. **(A)** Images of transiently transformed tobacco leaves 5 d after agroinfiltration. Four different assays are indicated: (1) empty vector, (2) *RsTT8* (bHLH), (3) *RsMYB1* (MYB), and (4) *RsTT8* and *RsMYB1*. **(B)** Anthocyanin contents. **(C)** Relative expression levels of endogenous anthocyanin structural and regulatory genes in tobacco plants, as determined by qPCR analysis. Results represent mean values ± SD from three biological replicates. ^*^, ^**^, and ^***^ indicate values that differ significantly from the control at *P* < 0.05, *P* < 0.01, and *P* < 0.001, respectively, according to Student's paired *t*-test.

To confirm the role of *RsTT8* and *RsMYB1* in anthocyanin biosynthesis, we conducted expression assays by infiltrating tobacco leaves with Agrobacterium strains harboring *RsMYB1* and *RsTT8* (Figure 5A). We found that RsTT8 alone did not trigger anthocyanin accumulation in tobacco leaves, similar to the mock infiltration control. By contrast, RsMYB1 alone and RsMYB1 coexpressed with RsTT8 resulted in red pigmentation of the infiltrated tobacco leaves. Red pigmentation appeared as early as 2 days post infiltration (dpi) and gradually accumulated up to 7 dpi. We measured the anthocyanin contents in leaf discs collected at 5 dpi. Anthocyanin levels were barely detectable in leaves infiltrated with mock control and RsTT8, were higher in leaves infiltrated with RsMYB1, and were highest in leaves coexpressing RsTT8 and RsMYB1 (Figure 5B). These results indicate that coexpression of RsTT8 and RsMYB1 stimulates anthocyanin accumulation compared with expression of RsMYB1 alone, and suggest that RsTT8 is a member of the MBW complex that regulates anthocyanin biosynthesis together with RsMYB1.

To confirm the relationship between expression levels of anthocyanin biosynthesis genes and anthocyanin contents, we analyzed the expression of ten structural genes involved in anthocyanin biosynthesis in the infiltrated tobacco leaves by qPCR. The genes examined included the upstream genes, *NtPAL* and *Nt4CL*, the EBGs *NtCHS, NtCHI, NtF3H, NtF3*′*H*, and *NtFLS*, and the LBGs *NtDFR, NtANS*, and *NtUFGT* (Figure 5C). Infiltration of RsTT8 alone did not induce expression of any of the anthocyanin biosynthesis genes, similar to that of the mock control. Infiltration of RsMYB1 alone increased the expression levels of most of the tested genes, except for *NtPAL, Nt4CL*, and *NtFLS*. Increased expression levels were observed for four of the EBGs examined (*NtCHS, NtCHI, NtF3H*, and *NtF3*′*H*) and all three LBGs (*NtDFR, NtANS*, and *NtUFGT*). Coinfiltration of RsTT8 with RsMYB1 substantially increased the expression levels of the four EBGs and all three LBGs. Increased expression of *NtAN2*, a bHLH TF, was only observed after coinfiltration of RsTT8 and RsMYB1. These gene expression patterns are consistent with the measured anthocyanin contents and red pigmentation, indicating that RsTT8 enhances anthocyanin biosynthesis.

### Protein–protein interactions between RsMYB1 and RsTT8

**Figure 6:**
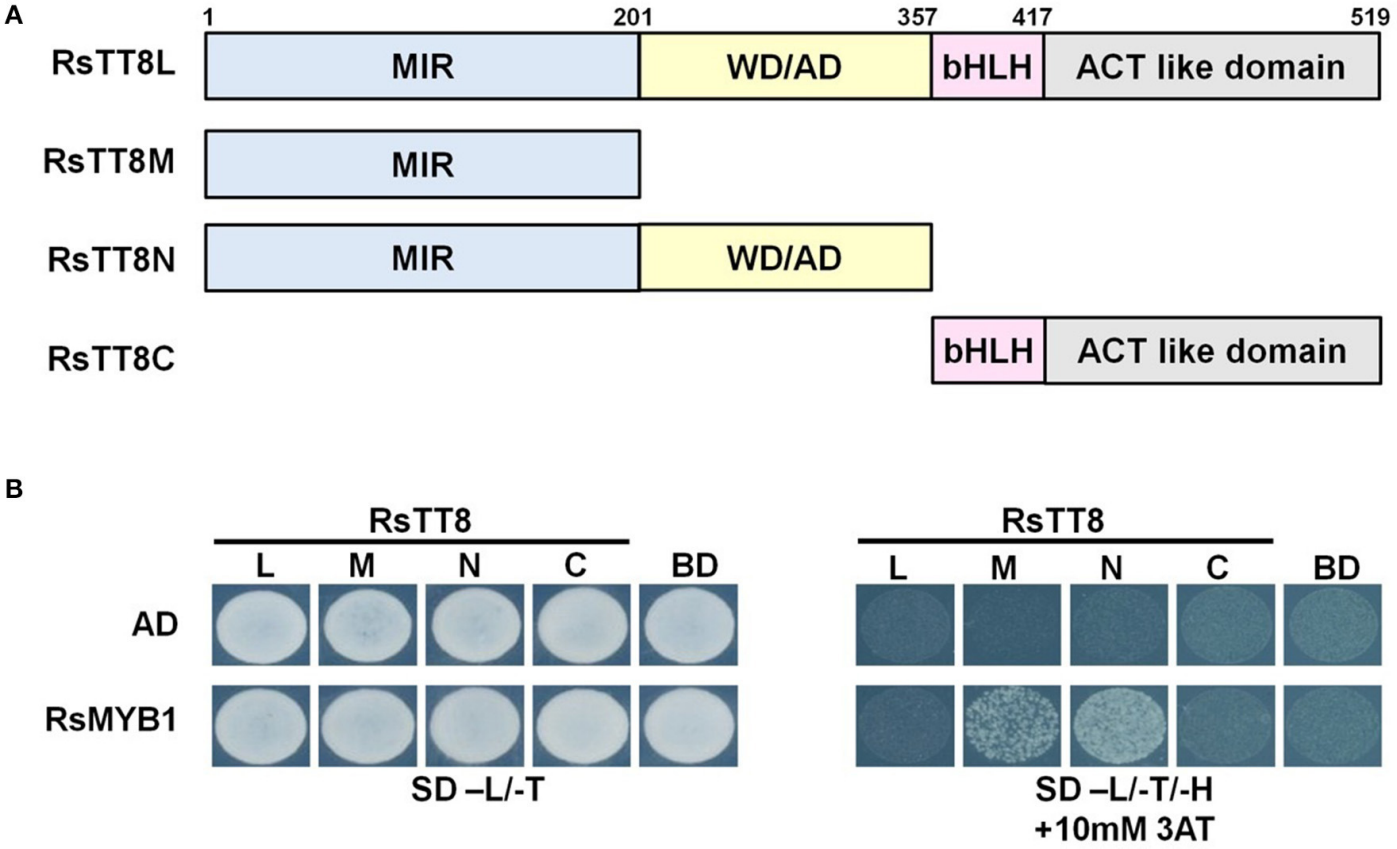
Physical interaction between RsTT8 and RsMYB1. **(A)** Schematic of full-length and partially truncated RsTT8. **(B)** Yeast two-hybrid analysis of interactions between RsMYB1 and different RsTT8 truncations. SD, minimal medium; AD, activation domain only; BD, binding domain only; 3AT, 3-amino-1,2,4-triazole; L, leucine; T, tryptophan; H, histidine.

The MBW complex regulates the expression of genes involved in anthocyanin biosynthesis. To investigate the interaction between RsMYB1 and RsTT8, we constructed four bait vectors encoding different RsTT8 regions (i.e., full-length RsTT8, and partially truncated RsTT8 fragments designated as RsTT8L, RsTT8M, RsTT8N, and RsTT8C) fused to the BD. These bait vectors were cotransformed into yeast strain AH109 along with the prey vector AD/RsMYB1. Yeast colonies expressing RsMYB1 and C-terminal RsTT8 truncations [RsTT8M (truncated protein including the MIR domain) and RsTT8N (truncated protein including the MIR and WD/AD domains)] grew on selection medium (–His–Leu–Trp) containing 10 mM 3AT, indicating strong protein–protein interactions between RsMYB1 and RsTT8 (Figure 6). By contrast, yeast colonies expressing RsMYB1 and RsTT8 constructs containing the C-terminal region [RsTT8L (full-length protein) and RsTT8C (truncated protein including bHLH and ACT-like domains)] did not grow on selection medium (–His–Leu–Trp) containing 10 mM 3AT, indicating that the C-terminal region was not essential for protein–protein interactions between RsMYB1 and RsTT8. These results confirm that the N-terminal MIR domain is essential for protein–protein interactions between RsTT8 and RsMYB1, and suggest that RsTT8 is a component of the MBW complex involved in anthocyanin biosynthesis.

### RsMYB1 and RsTT8 coregulate *RsCHS* and *RsDFR* promoter activity

**Figure 7:**
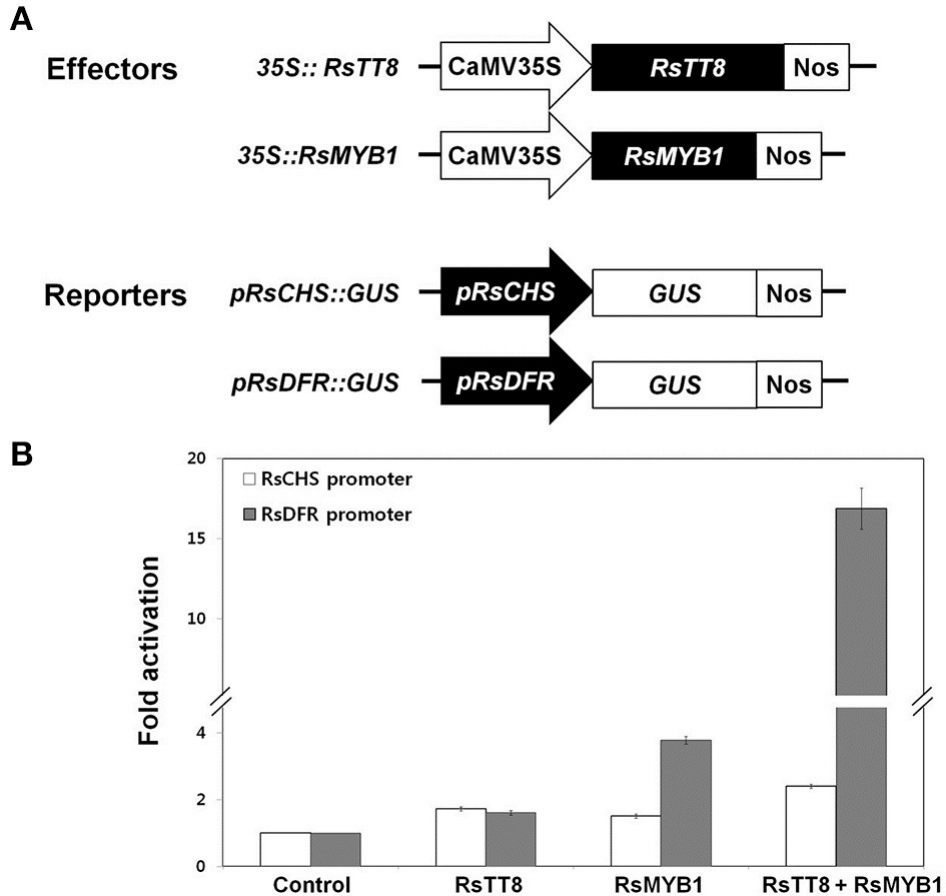
Effect of RsTT8 and RsMYB1 on *RsCHS* and *RsDFR* promoter activities. **(A)** Effector and reporter constructs used in this transcriptional activation assay. The effector construct contains *RsTT8* and *RsMYB1* driven by the CaMV 35S promoter. The reporter construct contains the *GUS* reporter gene driven by *RsCHS* and *RsDFR* promoters. **(B)** Transcriptional activation analysis of RsTT8- and RsMYB1-mediated induction of *RsCHS* and *RsDFR* promoter activities. A GUS reporter construct was used as the control, and GUS expression levels were set to 1. Results represent mean values ± SD from three biological replicates.

Anthocyanin biosynthesis is controlled by various TFs in the anthocyanin biosynthesis pathway (Hichri et al., 2011; Xu et al., 2015). The proximal promoter regions of genes involved in anthocyanin biosynthesis commonly contain a 7-bp MYB-recognizing element (MRE) and a 6-bp bHLH-recognizing element (BRE), and these elements are believed to be the targets of MYB and bHLH TFs, respectively (Zhu et al., 2015). Our results suggest that RsMYB1 and RsTT8 coregulate anthocyanin biosynthesis (Figures 4, 5). We thus examined whether these TFs could activate the expression of *RsCHS* and *RsDFR*, which displayed higher expression levels in anthocyanin-accumulating radish tissues and contained a number of MREs and BREs in their promoters (Supplementary Figure 1), using a transient transactivation system. RsTT8 and RsMYB1 were independently infiltrated or coinfiltrated into tobacco leaves along with modified pTr-GUS constructs containing the target *RsCHS* and *RsDFR* promoters driving *GUS* expression. We found that *RsTT8* alone did not activate the *RsCHS* and *RsDFR* promoters (Figure 7). RsMYB1 alone did not activate the *RsCHS* promoter, but did activate the *RsDFR* promoter (3.5-fold compared with the control). Coinfiltration of both RsTT8 and RsMYB1 resulted in approximately 2.5-fold and 18-fold increases in the promoter activities of *RsCHS* and *RsDFR*, respectively. These results indicate that RsMYB1-mediated activation of *RsCHS* and *RsDFR* promoter is enhanced by the bHLH TF RsTT8.

### RsTT8 functions in proanthocyanidin and anthocyanin biosynthesis

**Figure 8:**
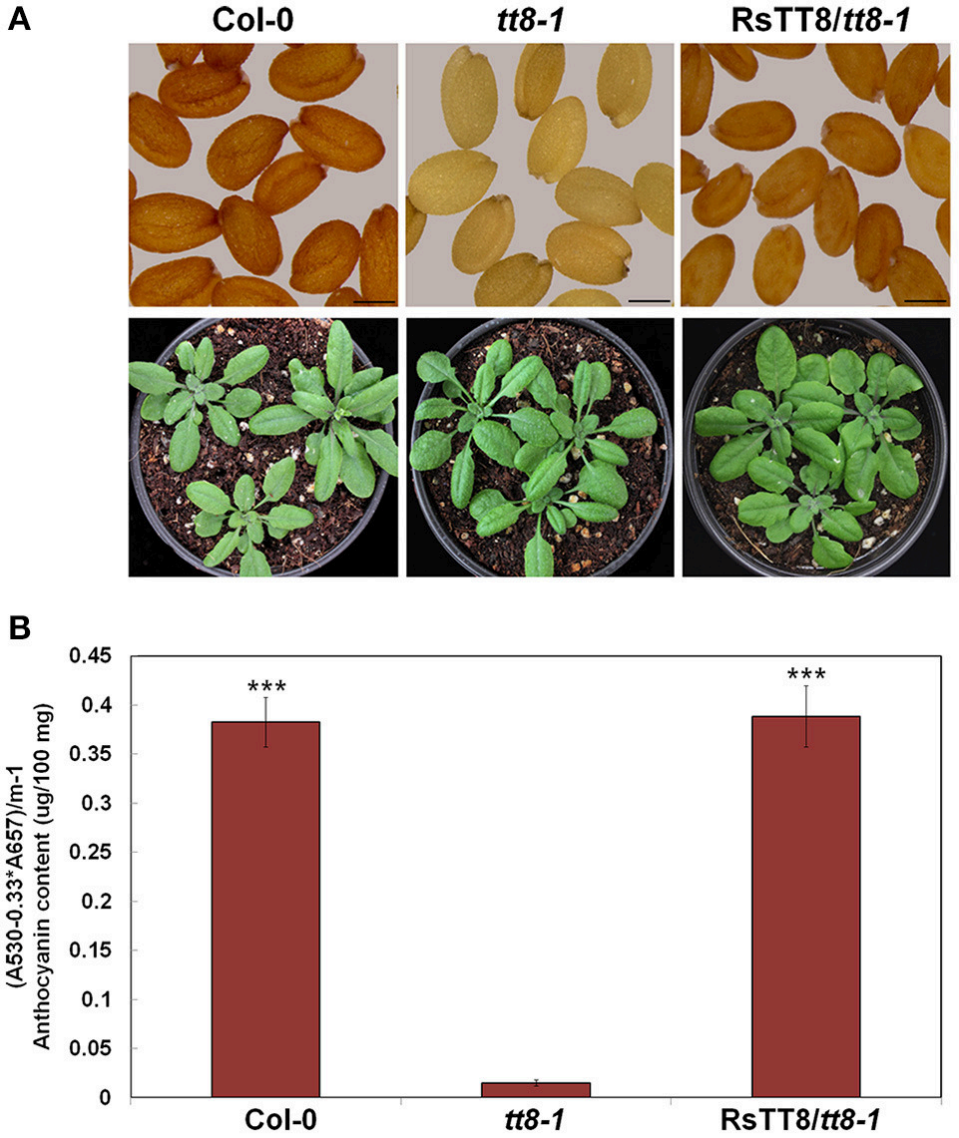
Phenotypes and anthocyanin contents in T_2_ transgenic Arabidopsis plants. **(A)** Phenotypic changes in *tt8-1* mutants expressing *RsTT8*. Seeds (top) and 3-week-old seedlings of wild-type Col-0 (left), *tt8-1* mutant (middle), and T_2_ progeny of *tt8-1* homozygotes transformed with *RsTT8*. **(B)** Anthocyanin contents in 3-week-old Arabidopsis seedlings. ^***^Indicates the value that differs significantly from the *tt8-1* mutant at *P* > 0.001 according Student paired *t*-test.

We next tested the effect of RsTT8 on the flavonoid biosynthetic pathway by expressing RsTT8 in the Arabidopsis *tt8-1* mutant (SALK_030966), which is deficient in PA, resulting in yellow seeds, and lacks anthocyanin in the junction between the stem and rosette leaves (Figure 8A). The T_2_ seed progeny originating from 18 independent T_1_ Basta-resistant transformants produced brown seeds similar to those of wild-type Arabidopsis. At the vegetative stage, T_3_ transgenic Arabidopsis displayed purple pigment in the junction between the stem and rosette leaves, indicating that the *tt8-1* mutant phenotype was complemented by RsTT8 expression. Next, we extracted pigments from whole leaves of wild-type, *tt8-1*, and *tt8-1* plants complemented with RsTT8 (Figure 8B). The anthocyanin content of tt8-1 was 4% that of wild-type plants, whereas the anthocyanin content of the complemented *tt8-1* plants was similar to that of wild-type plants, which is consistent with the coloration of the different lines. These results suggest that RsTT8 regulates PA biosynthesis in seeds and anthocyanin biosynthesis in leaves and stems.

## Discussion

### RsTT8 is a bHLH transcription factors that regulate anthocyanin biosynthesis

Anthocyanin biosynthesis is spatiotemporally regulated by MYB and bHLH TFs in various plants (Hichri et al., 2011; Xu et al., 2015). While RsMYB1 is known to be a key regulator of anthocyanin biosynthesis in radish, no bHLH TFs have been reported in this species to date (Lim et al., 2016a). In this study, we showed that RsTT8 is a bHLH transcription factor that acts with RsMYB1 to positively regulate anthocyanin biosynthesis. Specifically, we demonstrated that *RsTT8* and *RsMYB1* display similar expression patterns in anthocyanin-accumulating tissues of radish. The transcript levels of the anthocyanin biosynthesis genes *RsCHS* and *RsDFR* were significantly associated with the levels of *RsTT8* and *RsMYB1* in leaves, root flesh, and root skin, and also with anthocyanin content. Phylogenetic analysis showed that RsTT8 contained domains that are conserved in bHLH TFs. Specifically, the deduced amino acid sequence of RsTT8 contains MIR, AD/WD, bHLH, and ACT domains (Supplementary Figure 2). The bHLH domain of RsTT8 contains approximately 60 amino acids of a highly conserved HER motif (His9, Glu13, and Arg17). The HER motif has been identified in bHLH TFs known to regulate anthocyanin biosynthesis in several plant species, and has been shown to mediate binding to the E-box (CANNTG) DNA motif (Hichri et al., 2011; Xu et al., 2015). A dual luciferase assay showed that the activity of the morning glory (*Ipomoea purpurea*) *IpDFR-B* promoter was reduced when the E-box motif was mutated (Xu et al., 2015). Phylogenetic analysis indicated that RsTT8 belongs to the TT8 clade of the bHLH IIIf subgroup, clustering with bHLHs of Arabidopsis, cabbage, and rapeseed (i.e., members of the *Brassicaceae* family).

The MBW complex controls anthocyanin biosynthesis by regulating the transcription of structural genes in the anthocyanin biosynthesis pathway. In addition, the MBW controls many other physiological processes, such as trichome patterning and seed mucilage production. Y2H analyses previously showed that bHLH proteins such as AtTT8 and AtEGL interact with anthocyanin-activating MYBs, including AtPAP1, AtPAP2, AtMYB113, and AtMYB114 (Zimmermann et al., 2004). In this study, we showed that the N-terminal region of RsTT8 (i.e., RsTT8M and RsTT8N) interacts with RsMYB1, which is a key regulator of anthocyanin biosynthesis, whereas the C-terminal region of RsTT8 (i.e., RsTT8C and RsTT8L) inhibited the interaction (Figure 6). This result suggests that the MIR domain in the RsTT8 N-terminal region is indispensable for the interaction with RsMYB1.

### RsTT8 and RsMYB1 interact to regulate anthocyanin biosynthesis

We found that the expression profiles of *RsTT8* and *RsMYB1* and of the anthocyanin biosynthesis pathway genes *RsCHS, RsDFR*, and *RsANS* were positively associated with the patterns of anthocyanin accumulation in radish leaves and roots (Figure 4). In the transient assay, ectopic expression of *RsMYB*1 in tobacco induced the high expression of *NtAN1* gene that was overlapping the expression pattern of anthocyanin biosynthetic pathway genes (Figure 5). These results were consistent with previous work that bHLH and MYB proteins have essential roles in anthocyanin biosynthesis (Liu et al., 2016; Li et al., 2017). Interestingly, the transcript level of *RsMYB1* and *RsTTG1* was the highest, but that of *RsTT8* was lower in white root flesh (D variety) as a similar result with white potato flesh (Liu et al., 2016). This result suggests that RsTT8 is a limiting regulator in anthocyanin biosynthesis in flesh.

Hierarchical regulation between bHLH TFs of MBW complex has been reported in some plants including Arabidopsis, freesia, petunia and tobacco (Albert et al., 2014; Montefiori et al., 2015; Xiang et al., 2015; Li et al., 2016; Liu et al., 2016). In Arabidopsis, bHLH TF AtTT8 was activated by MBW complex with AtTT8 itself or with other bHLH factors, AtGL3 and AtEGL3. Unlike Arabidopsis, PhAN1 could not be activated its own expression, while it was activated by PhJAF13. Through the promoter activation assay, it showed that NtJAF13 was involved in the transcriptional activation in NtAN1, which lead to regulate the anthocyanin biosynthetic genes. Similar hierarchical expression pattern between FhGL3L and FhTT8L was also observed in freesia. It seems to be the common regulatory network between bHLH TFs of MBW complex for anthocyanin biosynthesis.

Activation of RsCHS and RsDFR expression was controlled by RsMYB1 and enhanced by the presence of RsTT8 (Figures 5, 7). A previous study reported that MYB and bHLH targeted cis elements in anthocyanin biosynthesis pathway genes (Zhu et al., 2015). The promoter of *RsCHS* contains four MREs and five BREs, while that of *RsDFR* contains three of these elements. Whereas these elements are widely distributed throughout the promoter region of *RsCHS*, they are close to the translational start site in *RsDFR* (Supplementary Figure 1). The cis architecture determines the promoter activity (Zhu et al., 2015). Coexpression of RsTT8 and RsMYB1 in tobacco leaves increased the promoter activity of *RsDFR* to a greater extent than that of *RsCHS*. Previous studies of Zhu et al. (2015), it showed that the BREs and MREs located in the proximal regions typically within 350 bp from the translation start site of anthocyanin biosynthetic pathway genes. Through analysis with the 159 promoters of 35 species, it found that pairing between BRE and MRE was within 100 bp of each other more than half (53%) including *Ginkgo biloba*, monocots and dicots. In addition, the artificially increasing the distance between BREs and MREs significantly weakened the promoter activity strength. Taken together these results, it suggests that the *cis* element location is more important than the number of *cis* elements.

Several studies have reported that more than one bHLH TF regulates anthocyanin and PA biosynthesis in plants (e.g., AtTT8, AtGL3, and AtEGL3 in Arabidopsis (Nesi et al., 2000; Payne et al., 2000; Zhang et al., 2003), FhTT8L and FhGL3L in freesia (Li et al., 2016), PhAN1 and PhJAF13 in petunia (Quattrocchio et al., 1998; Spelt et al., 2000), and NtAn1 and NtJAF13 in tobacco (Bai et al., 2011; Montefiori et al., 2015). These bHLH TFs may have overlapping functions in anthocyanin biosynthesis, or they may interact with specific MYB TFs to perform specialized functions, such as PA synthesis, seed coat mucilage production, and initiation of trichomes and root hairs (Petroni and Tonelli, 2011). We observed that RsTT8 has overlapping roles in PA biosynthesis in seeds and anthocyanin accumulation in leaves and stems (Figure 8). Transgenic tobacco leaves coexpressing RsTT8 and RsMYB1 accumulated significantly higher levels of anthocyanin than did those expressing RsMYB1 or RsTT8 alone (Figure 5). Furthermore, anthocyanin accumulation was associated with up-regulation of *NtAN1*, the endogenous bHLH TF. Previous studies reported that expression of exogenous MYB in tobacco led to enhanced anthocyanin accumulation if endogenous NtAN1 expression was enhanced (Montefiori et al., 2015; Liu et al., 2016; Chen et al., 2017). However, transgenic petunia containing MdMYB10_R6_ had colored flower and showed the absence of pigment accumulation in vegetative tissues due to the failure to form a functional MBW complex for anthocyanin biosynthesis (Boase et al., 2015). The phenotypic differences in pigment accumulation within the transgenic petunias may be due to distinction in the availability and binding capacity of endogenous bHLH and WDR. Thus, it is necessary to form an active complex through interaction between exogenous and endogenous TFs for manipulating the production of valuable secondary metabolites in plants.

Taken together these results, simultaneous expression of bHLH and MYB TFs is very critical for anthocyanin accumulation via the activation of antocyanin biosynthetic pathway genes. Further studies on hierarchical regulation and feedback regulation mechanisms of MBW complex will provide insight into spatiotemporally regulating anthocyanin biosynthesis in plant.

## Conclusion

This work shows that the bHLH TF RsTT8 functions in anthocyanin biosynthesis in radish. RsTT8 belongs to the TT8 clade of the bHLH group IIIf proteins, and interacts with the MIR domain of RsMYB1 to form a presumed MBW complex. Coexpression of RsTT8 and RsMYB1 induces anthocyanin accumulation and upregulates the expression of genes in the anthocyanin biosynthesis pathway. Complementation tests indicate that RsTT8 also functions in PA biosynthesis in seeds.

## Materials and methods

### Plant materials

Radish (*Raphanus sativus* L.) seeds were obtained from the Agricultural Genetic Resources Center at the National Academy of Agricultural Science (Jeonju, Korea). The following three radish cultivars were used in this study: Cheongpihongsim (C, IT100676), Daelyughonghwan (D, IT100675), and Namwonammu (N, IT102388). The radish cultivars were grown in greenhouses and photographed at the mature stage (6 weeks old). Quantitative real-time polymerase chain reaction (qPCR) and anthocyanin content analyses were conducted using mature leaves and the skins and flesh of mature roots. Transformation experiments were conducted using *Arabidopsis thaliana* ecotype Columbia-0 and the transparent testa (*tt*) mutant line *tt8-1* (SALK_030966), which was obtained from the Arabidopsis Biological Resource Center (ABRC). All of these plants were grown on Murashige and Skoog (MS) medium or in soil under long-day conditions (LD, 16-h light/8-h dark, 100 μmol m^−2^s^−1^) at 22°C. Transient transformation experiments were conducted using tobacco (*Nicotiana tabacum*) plants, which were grown in greenhouses under natural light at 28°C.

### Cloning of *RsTT8* from red radish plants

Total RNA was extracted from the leaves of red radishes [homozygotic F_3_ progeny of “Bordeaux” (Syngenta, Co.)] using TRIzol reagent (Invitrogen, Carlsbad, CA) according to the manufacturer's instructions (Lim et al., 2016a). The full-length cDNA of the radish *TT8* gene was obtained by performing 5′-rapid amplification of cDNA ends (RACE) and 3′-RACE using the SMART RACE cDNA Amplification Kit (Clontech, Madison, WI) with four gene-specific primers (3′race-TT8-F1, 3′race-TT8-F2, 5′race-TT8-R1, and 5′race-TT8-R2), which were designed on the basis of the partially sequenced radish *TT8* gene (accession no. JN625953.1). An additional primer set (RsTT8-F/RsTT8-R) was prepared to amplify the full-length cDNA gene. All PCR fragments were subcloned into the pGEM-T Easy vector (Promega, Madison, WI) or the pENTR/D-TOPO vector (Invitrogen) to validate DNA sequences. All primer sequences are listed in Supplementary Table 1.

### Bioinformatics analysis

The nucleotide sequence, deduced amino acid sequence, and open reading frame (ORF) of *RsTT8* were subjected to BLAST analysis on the National Center for Biotechnology Information (NCBI) website (http://www.ncbi.nlm.nih.gov). Structural analysis of the deduced protein was conducted using the ExPASy Molecular Biology Server (https://www.expasy.org/tools). Multiple sequence alignments were performed using CLUSTAL W (Thompson et al., 1994). A phylogenetic tree was constructed with the neighbor-joining method (Saitou and Nei, 1987) using MEGA version 6 software (Kumar et al., 2001). *Cis*-elements in the *RsCHS* and *RsDFR* promoters were analyzed according to the method of Zhu et al. (2015).

### RsTT8 subcellular localization assay

The subcellular localization of RsTT8 was analyzed in Arabidopsis protoplasts as described by Yoo et al. (2007). GFP fusion constructs were generated in p326-sGFP plasmid, which contains a cauliflower mosaic virus 35S promoter. For the C-terminal GFP fusion, the ORF of RsTT8 was amplified using gene-specific primer sets (p326-RsTT8-F/R), which introduced an *Xba*I site upstream of the ATG codon with the InFusion Cloning System (Clontech). The resultant p326-RsTT8-sGFP plasmid was sequenced to confirm the absence of errors during PCR amplification. Plasmids were introduced into Arabidopsis protoplasts prepared from leaf tissues using polyethylene glycol-mediated transformation. Fusion construct expression was monitored 16–20 h after transformation, and images were captured by fluorescence confocal microscopy (Leica TCS SP8, Leica Microsystems, Germany).

### Quantitative real-time polymerase chain reaction (qPCR) analysis

Total RNA from radish and tobacco leaves was prepared using TRIzol Reagent (Invitrogen), and first-strand cDNAs were generated using the cDNA EcoDry Kit (Clontech). The qPCR conditions and gene-specific primers, with the exception of those for tobacco *NtAN1* and *NtAN2*, were as described in previously (Lim et al., 2016a,b). The qPCR primers for *NtAN1* and *NtAN2* were as follows: *NtAN1* (F, 5′-ACCATTCTCGAACACCGAAG-3′; R, 5′-TGCTAGGGCACAATGTGAAG-3′) and *NtAN2* (F, 5′-GTAGACTTCCTGGAAGGACGGCAA-3′; R, 5′-GGCCGAGGTCTGAATATGGTGATC-3′). Gene expression was normalized using the RNA polymerase-II (*RPII*) and glyceraldehyde 3-phosphate dehydrogenase (*GAPDH*) genes for radish and tobacco, respectively, as internal references. Three biological replicates were examined for each sample.

### Measurement of total anthocyanin content

Total anthocyanin content was determined according to the method described by Shin et al. (2007). Briefly, powdered tissue samples were incubated in 600 μL extraction buffer (methanol containing 1% HCl) for 6 h at 4°C with moderate shaking. Then, 200 μL water and 200 μL chloroform were added, followed by centrifugation at 14,000 g for 5 min at 4°C to sediment the plant material. The absorbance of the supernatant was recorded at 530 nm (A_530_) and 657 nm (A_657_) using a microplate reader. Anthocyanin content was determined using the following equation: A_530_ − 0.33 × A_657_. The anthocyanin content in each sample was measured in three independent experiments.

### Yeast two-hybrid (Y2H) assays

For Y2H experiments, we used the pGADT7 and pGBKT7 vectors harboring the GAL4 activation domain (AD) and GAL4 DNA-binding domain (BD) (Clontech). The full-length *RsMYB1* cDNA was inserted into pGADT7 vector, generating the AD-RsMYB1 construct. To generate *RsTT8* binding-domain (BD) constructs, the following regions were amplified using specific primer sets: full-length *RsTT8* (RsTT8L), MYB-interacting region (MIR domain), truncated protein including the MIR domain (RsTT8M), N-terminal region, truncated protein including the MIR and WD/AD domains (RsTT8N) and C-terminal region, truncated protein including bHLH and ACT-like domains (RsTT8C) (Supplementary Table 1). The amplified fragments were ligated into the pBD vector (Stratagene). AD and BD constructs were cotransformed into yeast strain AH109 according to the manufacturer's instructions (Stratagene). Yeast strains were selected on SD media lacking Leu and Trp and were replicated on SD media lacking Leu, Trp, and His and supplemented with 10 mM 3-amino-1,2,4-triazole (3AT), a competitive inhibitor of the *HIS3* gene product. Growth was scored after 2 d at 30°C.

### In planta assay of RSTT8 function

The plasmid used for transient transformation of tobacco and stable transformation of Arabidopsis was constructed as follows. The *RsTT8* ORF was subcloned into the pENTR/D-TOPO vector (Invitrogen) and incorporated into the Gateway destination vector pB7WG2D (VIB-Ghent University, Ghent, Belgium) through several Gateway cloning steps. The resultant vector was maintained in *Agrobacterium tumefaciens* strain GV3101 and infiltrated into the abaxial surfaces of *Nicotiana tabacum* leaves. Leaf color was monitored at 5 days post-infiltration (dpi) as described by Lim et al. (2016a).

The pB7WG2D-RsTT8 construct was maintained in Agrobacterium strain GV3101 and transformed into the Arabidopsis *tt8-1* mutant (SALK_030966) using the floral dipping method. Transformed Arabidopsis seeds were grown in soil under 16-h light/8-h dark conditions at 20°C. Transgenic Arabidopsis plants were selected by spraying the plants with 0.3% Basta solution. Homozygous T_2_ lines were selected and used for further analysis.

### Promoter activation assay

To isolate the *RsCHS* (accession number: LOC108843267) and *RsDFR* (accession number: LOC108843267) promoters, specific primers were designed based on the radish whole-genome sequence (Supplementary Table 1). Conserved *cis*-element motifs located in the promoters are illustrated in (Supplementary Figure 1). The reporter fusion construct was generated by inserting the *RsCHS* and *RsDFR* promoters into the pTr-GUS vector at the 5′ end of the *GUS* gene (derived from pBI121) after removing the CaMV35S promoter region. The resultant constructs were transferred into the plant expression vector pBAR, which was generated by *Pst*I digestion of pB7WG2D. The ORFs of RsTT8 and RsMYB1 were incorporated into the Gateway destination vector pB7WG2D through several Gateway cloning steps. The resultant constructs, pB7WG2D-RsTT8 and pB7WG2D-RsMYB1 were used as effector constructs. The all resultant vectors were transformed into Agrobacterium strain GV3101.

Transient promoter activation assays were performed in *N. tabacum* as follows. Agrobacteria containing reporter and effector constructs were cultured in LB media for 2 d at 28°C, pelleted by centrifugation at 6,000 rpm for 5 min at 4°C, resuspended in infiltration buffer (10 mM MgCl_2_ and 100 μM acetosyringone) to an OD_600_ of 0.2 (approximately 10 ml of buffer), and incubated at room temperature without shaking for 2 h. Before infiltration into tobacco leaves, Agrobacteria harboring effector and reporter constructs were mixed at a ratio of 3:1, respectively. Tobacco leaves were infiltrated with Agrobacteria harboring the genes of interest, and then the leaves were harvested to assay GUS activity at 3 days post-infiltration (dpi) as described in Lim et al. (2013). Agrobacteria harboring only the GUS reporter construct were used as the control. At least three biological replicates were used for each experiment.

## Author contributions

S-HL designed and performed the experiments and prepared the figures. D-HK participated in qPCR analysis, Y2H assay and Arabidopsis transformation. JK analyzed the anthocyanins. J-YL carried out the transient assay of tobacco leaves. S-HH designed the study and wrote the manuscript with S-HL. All authors read and approved the final manuscript.

### Conflict of interest statement

The authors declare that the research was conducted in the absence of any commercial or financial relationships that could be construed as a potential conflict of interest.

## Supplementary material

Supplementary Figure 1 RsCHS and RsDFR promoter architectures. **(A)** Schematic of the RsCHS and RsDFR promoters showing putative bHLH- and MYB-interacting cis-elements involved in anthocyanin biosynthesis. The cis-elements are indicated by different symbols. **(B)** Nucleotide sequence of the RsCHS promoter. **(C)** Nucleotide sequence of the RsDFR promoter. BREs and MREs are indicated with red and green boxes, respectively, and the expected TATA box is shown in bold.

Supplementary Figure 2 Protein sequence alignments of RsTT8 and known anthocyanin-related bHLHs. MIR (MYB-interacting region), acidic WD/AD, bHLH, and ACT-like domains are shaded in different colors. The 19 conserved residues of the bHLH domain are represented using the red boxes. Arrows indicate the HER motif in the bHLH domain.

## Abbreviations

3AT: 3-amino-1,2,4-triazole
4CL: 4-coumarate-CoA ligase
AD: activation domain
ANS: anthocyanidin synthase
BD: binding domain
bHLH: basic helix-loop-helix
CHI: chalcone isomerase
CHS: chalcone synthase
DFR: dihydroflavonol 4-reductase
F3′H: flavonoid 3′ hydroxylase
F3H: flavanone 3-hydroxylase
FLS: flavonol synthase
GFP: green fluorescent protein
MBW: MYB-bHLH-WDR protein complex
PAL: phenylalanine ammonia lyase
RFP: red fluorescent protein
UFGT: UDP-glucose: flavonoid 3-O-glucosyltransferase
Y2H: yeast two hybrid assay.
