# Supplementary figures and images for: A Radish Basic Helix-Loop-Helix Transcription Factor, RsTT8 Acts a Positive Regulator for Anthocyanin Biosynthesis
Source: Front Plant Sci. 2017 Nov 8;8:1917. doi: 10.3389/fpls.2017.01917 (PMC5682339; doi:10.3389/fpls.2017.01917)

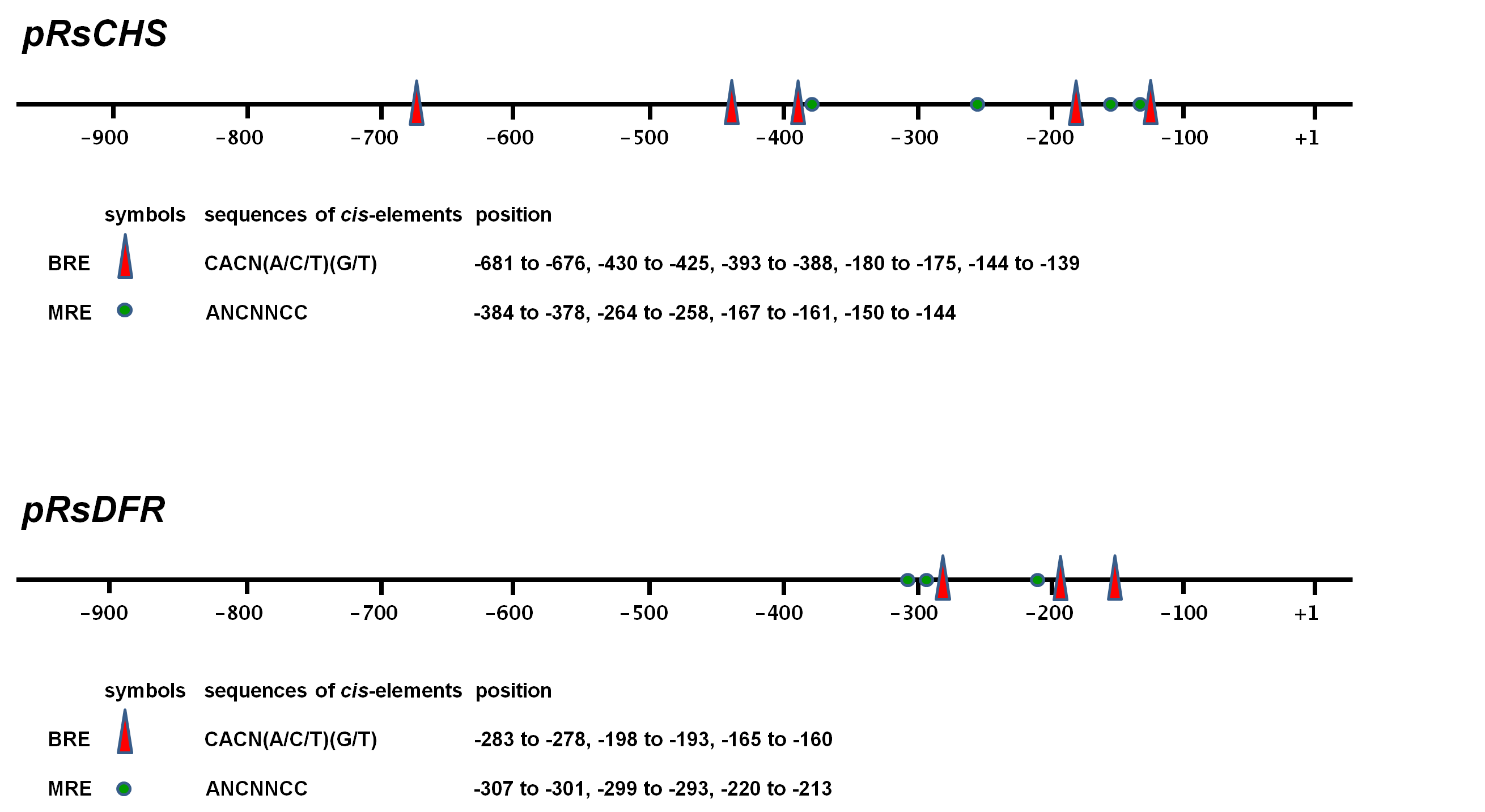

Supplement: Supplementary Figure 1 — RsCHS and RsDFR promoter architectures. (A) Schematic of the RsCHS and RsDFR promoters showing putative bHLH- and MYB-interacting cis-elements involved in anthocyanin biosynthesis. The cis-elements are indicated by different symbols. (B) Nucleotide sequence of the RsCHS promoter. (C) Nucleotide sequence of the RsDFR promoter. BREs and MREs are indicated with red and green boxes, respectively, and the expected TATA box is shown in bold. [file Image1.TIF]

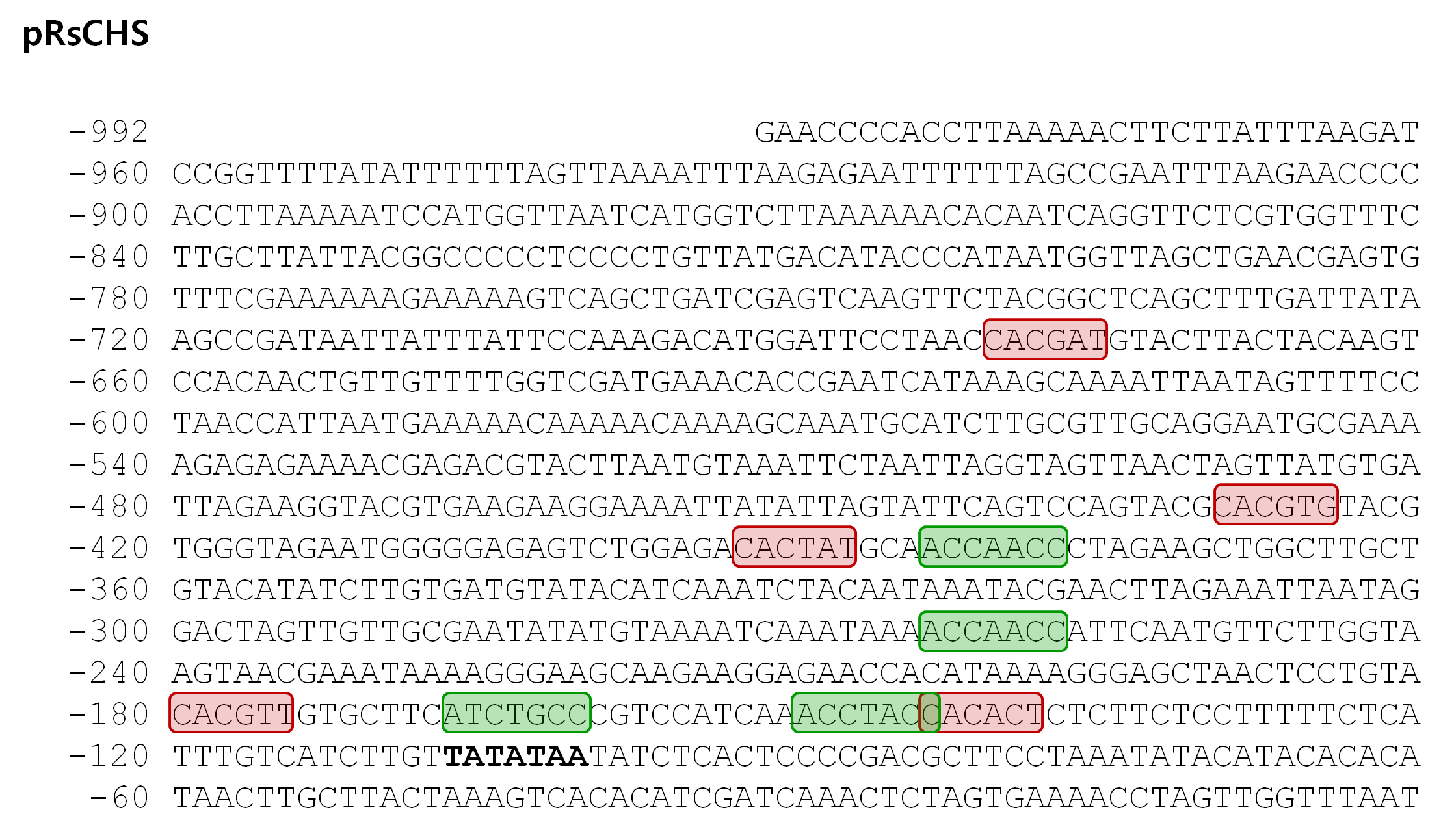

Supplement: Supplementary Figure 2 — Protein sequence alignments of RsTT8 and known anthocyanin-related bHLHs. MIR (MYB-interacting region), acidic WD/AD, bHLH, and ACT-like domains are shaded in different colors. The 19 conserved residues of the bHLH domain are represented using the red boxes. Arrows indicate the HER motif in the bHLH domain. [file Image2.TIF]

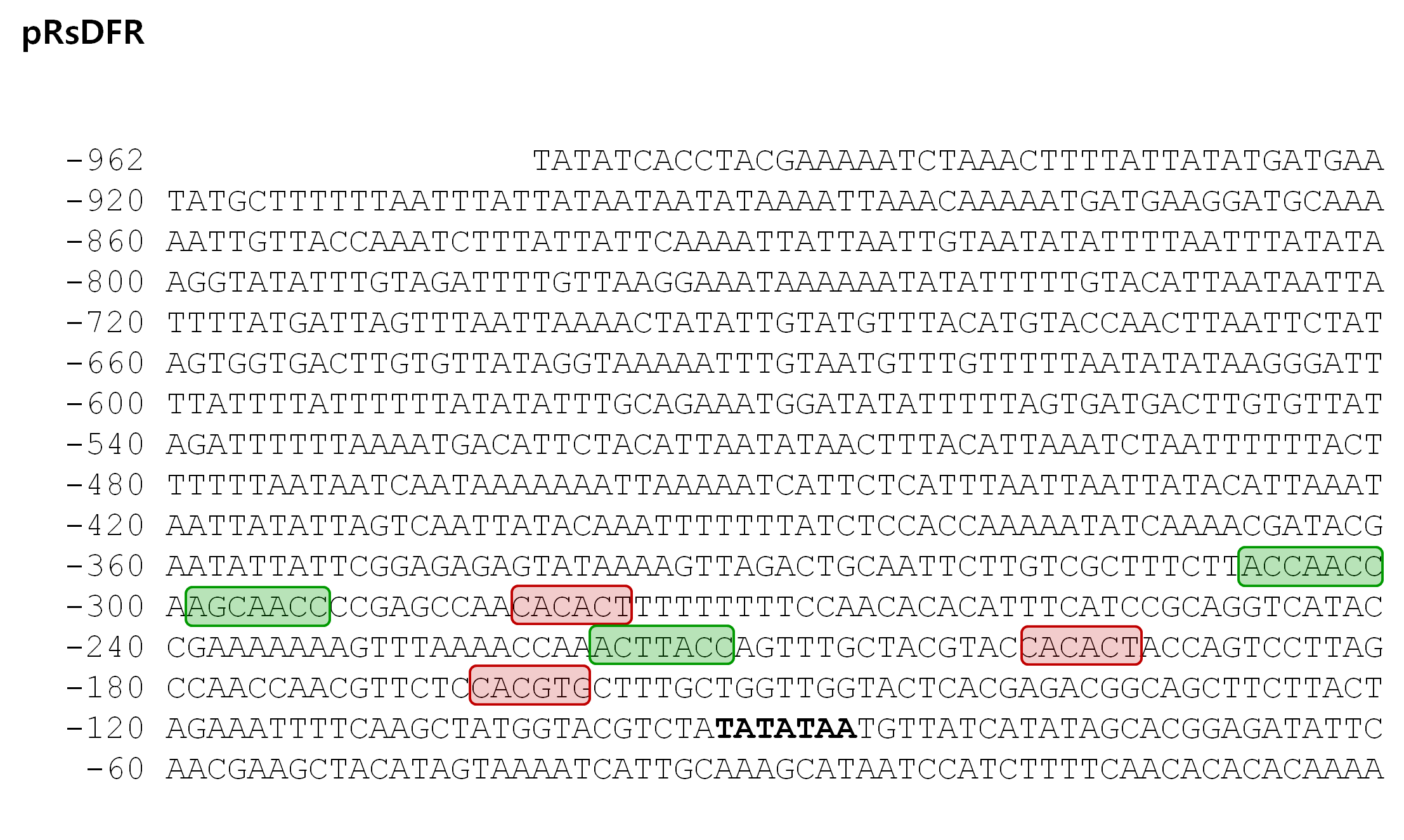

Supplement: Supplementary file 3 [file Image3.TIF]

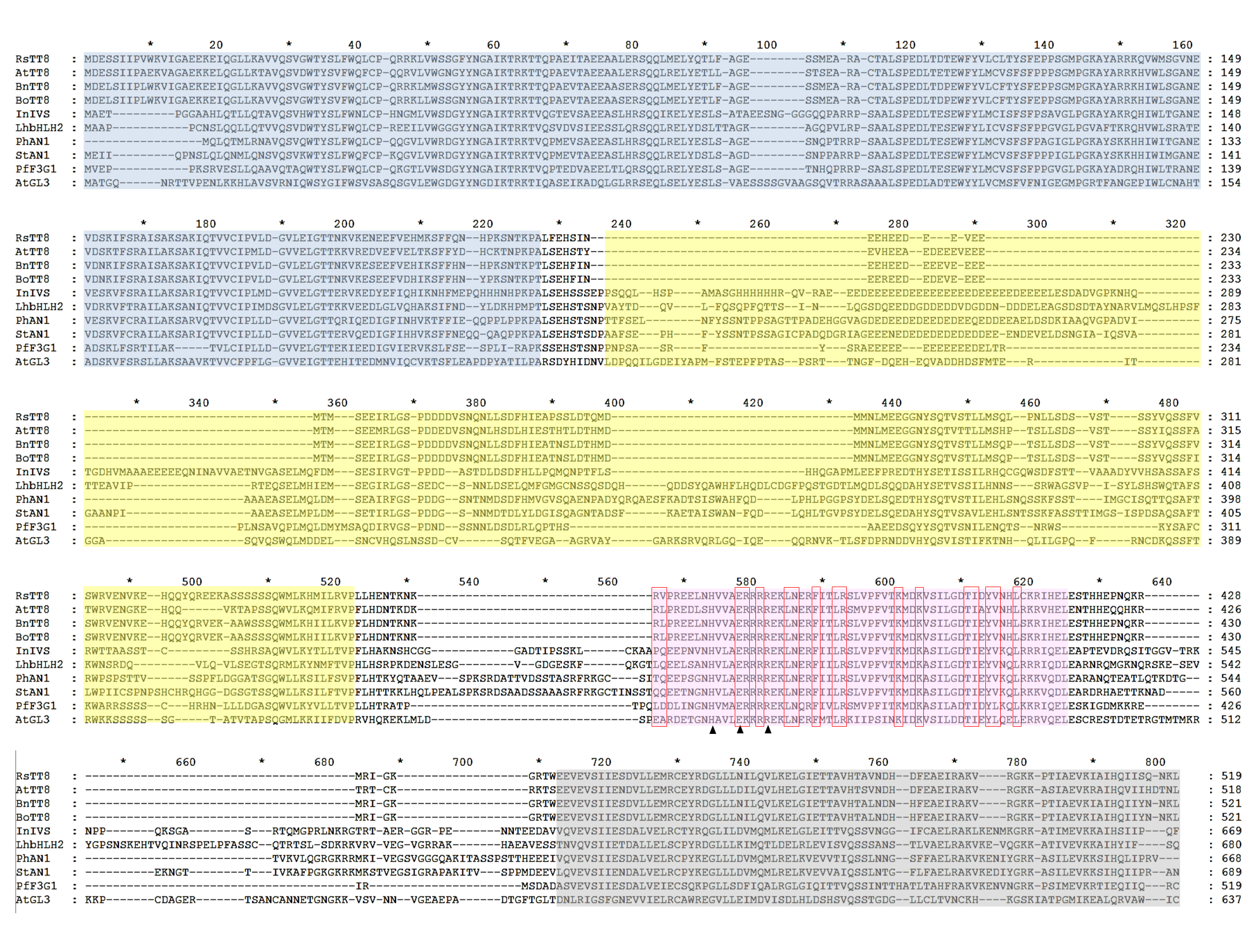

Supplement: Supplementary file 4 [file Image4.TIF]
