# Supplementary material for: A Radish Basic Helix-Loop-Helix Transcription Factor, RsTT8 Acts a Positive Regulator for Anthocyanin Biosynthesis
Source: Front Plant Sci. 2017 Nov 8;8:1917. doi: 10.3389/fpls.2017.01917 (PMC5682339; doi:10.3389/fpls.2017.01917)
Supplement: Supplementary file 5 [file Table1.docx]

**SupplementaryTable legends**

Supplementary Table 1. qRT-PCR primers used in this study

| Primer | Sequence | Usage |
| --- | --- | --- |
| 3’race-TT8-F1 | 5’-ggcggtggtgcaatctgtggggtggacttatag-3’ | 3’-RACE PCR |
| 3’race-TT8-F2 | 5’-ggaagcgagggcttgcacagcactgt-3’ | 3’-RACE PCR |
| 5’race-TT8-R1 | 5’-CCGCCTTCCTCCATTAGATTCATCATGTCC-3’ | 5’-RACE PCR |
| 5’race-TT8-R2 | 5’-GCTCTTCGTTGATGGAGTGTTCAAAAAGAGCAGG-3’ | 5’-RACE PCR |
| RsTT8-F | 5’-CACCATGGATGAATCAAGTATTATACCGGTATGG-3’ | Gene cloning |
| RsTT8-R | 5’-CTAGAGTTTATTTTGAGATATGATTTGATGG-3’ | Gene cloning |
| p326-RsTT8-F | 5’- CACGGGGGACTCTAGAATGGATGAATCAAGTATTATAC-3’ | GFP expression vector |
| p326-RsTT8-R | 5’- CCATGGATCCTCTAGAGAGTTTATTTTGAGATATGATTT-3’ | GFP expression vector |
| pBD-RsTT8-F | 5’- CATGGAGGCCGAATTCATGGATGAATCAAGTATTATAC-3’ | Yeast 2 hybrid assay |
| pBD-RsTT8-R | 5’- GGATCCCCGGGAATTCGAGTTTATTTTGAGATAT -3’ | Yeast 2 hybrid assay |
| pBD-RsTT8M-R | 5’-GGATCCCCGGGAATTCGAAGAAACTCTTCATGTGTTCAAC -3’ | Yeast 2 hybrid assay |
| pBD-RsTT8N-R | 5’-GGATCCCCGGGAATTCAGGAACTCTCAAGATCATGTGTTTG -3’ | Yeast 2 hybrid assay |
| pBD-RsTT8C-F | 5’-CATGGAGGCCGAATTCCATGAAGAAGACGAAGAAGTAG -3’ | Yeast 2 hybrid assay |
| pAD-RsMYB1-F | 5’-GGAGGCCAGTGAATTCATGGAGGGTTCGTCCAAAGGG-3 | Yeast 2 hybrid assay |
| pAD-RsMYB1-R | 5’-CACCCGGGTGGAATTCTTACACAGTCTCTCCATCTAACAGG-3’ | Yeast 2 hybrid assay |
| pRsCHS-F | 5’-GAACCCCACCTTAAAAACTTCTTA-3’ | Promoter isolation |
| pRsCHS-R | 5’-ATTAAACCAACTAGGTTTTCACTAG-3’ | Promoter isolation |
| pRsDFR-F | 5’-TATATCACCTACGAAAAATCTAAACTTTTA-3’ | Promoter isolation |
| pRsDFR-R | 5’-TTTTGTGTGTGTTGAAAAGATGGA-3’ | Promoter isolation |
| pTr-pRsCHS-GUS-F | 5’-GGCCAGTGCCAAGCTTGAACCCCACCTTAAAAACTTCTTA-3’ | Promoter activation assay |
| pTr-pRsCHS-GUS-R | 5’-GACCACCCGGGGATCCATTAAACCAACTAGGTTTTCACTAG-3’ | Promoter activation assay |
| pTr-pRsDFR-GUS-F | 5’-GGCCAGTGCCAAGCTTTATATCACCTACGAAAAATCTAAAC-3’ | Promoter activation assay |
| pTr-pRsDFR-GUS-R | 5’-GACCACCCGGGGATCCTTTTGTGTGTGTTGAAAAGATGGA-3’ | Promoter activation assay |
| pBAR-pRsCHS-F | 5’-CCGACGTCGCATGCCTGCAGGAACCCCACCTTAAAAACTTCTTA-3’ | Promoter activation assay |
| pBAR-pRsDFR-F | 5’-CCGACGTCGCATGCCTGCAGTATATCACCTACGAAAAATCTAAAC-3’ | Promoter activation assay |
| pBAR-Nos-R | 5’-CTAAGCTTGCATGCCTGCAGGAATTCCCGATCTAGTAACATAG-3’ | Promoter activation assay |
